# Transcatheter Aortic Valve Replacement with the Self-Expandable Core Valve Evolut Prosthesis Using the Cusp-Overlap vs. Tricusp-View

**DOI:** 10.3390/jcm11061561

**Published:** 2022-03-12

**Authors:** Philipp Maximilian Doldi, Lukas Stolz, Felix Escher, Julius Steffen, Jonas Gmeiner, Daniel Roden, Marie Linnemann, Kornelia Löw, Simon Deseive, Thomas J. Stocker, Martin Orban, Hans Theiss, Konstantinos Rizas, Adrian Curta, Sebastian Sadoni, Joscha Buech, Dominik Joskowiak, Sven Peterss, Christian Hagl, Steffen Massberg, Jörg Hausleiter, Daniel Braun

**Affiliations:** 1 Medizinische Klinik und Poliklinik I, Klinikum der Universität München, 81377 Munich, Germany; lukas.stolz@med.uni-muenchen.de (L.S.); julius.steffen@med.uni-muenchen.de (J.S.); jonas.gmeiner@med.uni-muenchen.de (J.G.); daniel.roden@med.uni-muenchen.de (D.R.); marie.linnemann@med.uni-muenchen.de (M.L.); kornelia.loew@med.uni-muenchen.de (K.L.); simon.deseive@med.uni-muenchen.de (S.D.); thomas.stocker@med.uni-muenchen.de (T.J.S.); martin.orban@med.uni-muenchen.de (M.O.); hans.theiss@med.uni-muenchen.de (H.T.); konstantinos.rizas@med.uni-muenchen.de (K.R.); steffen.massberg@med.uni-muenchen.de (S.M.); joerg.hausleiter@med.uni-muenchen.de (J.H.); daniel.braun@med.uni-muenchen.de (D.B.); 2 German Center for Cardiovascular Research (DZHK), Munich Heart Alliance, 80539 Munich, Germany; 3 Klinik und Poliklinik für Radiologie, Klinikum der Universität München, 81377 Munich, Germany; felix.escher@med.uni-muenchen.de (F.E.); adrian.curta@med.uni-muenchen.de (A.C.); 4 Herzchirurgische Klinik und Poliklinik, Klinikum der Universität München, 81377 Munich, Germany; sebastian.sadoni@med.uni-muenchen.de (S.S.); joscha.buech@med.uni-muenchen.de (J.B.); dominik.joskowiak@med.uni-muenchen.de (D.J.); sven.peterss@med.uni-muenchen.de (S.P.); christian.hagl@med.uni-muenchen.de (C.H.)

**Keywords:** TAVR, aortic stenosis, TAVI, permanent pacemaker implantation, conduction disturbance, self-expandable valves, cusp-overlap view, three-cusp view

## Abstract

Despite the rapid increase in experience and technological improvement, the incidence of conduction disturbances in patients undergoing transcatheter aortic valve replacement (TAVR) with the self-expandable CoreValve Evolut valve remains high. Recently, a cusp-overlap view (COP) implantation technique has been proposed for TAVR with self-expandable valves offering an improved visualization during valve expansion compared to the three-cusp view (TCV). This study aims to systematically analyze procedural outcomes of TAVR patients treated with the CoreValve Evolut valve using a COP compared to TCV in a high-volume center. The primary endpoint was technical success according the 2021 VARC-3 criteria. A total of 122 consecutive patients (61 pts. TCV: April 2019 to November 2020; 61 pts. COP: December 2020 to October 2021) that underwent TAVR with the CoreValve Evolut prosthesis were included in this analysis. Although there was no difference in the primary endpoint technical success between TCV and COP patients (93.4% vs. 90.2%, OR 0.65, 95% CI 0.16, 2.4, *p* = 0.51), we observed a significantly lower risk for permanent pacemaker implantation (PPI) among COP patients (TCV: 27.9% vs. COP: 13.1%, OR 0.39, 95% CI 0.15, 0.97, *p* = 0.047). Implantation of the CoreValve Evolut prosthesis using the COP might help to reduce the rate of PPI following TAVR.

## 1. Introduction

In patients with symptomatic severe aortic stenosis (AS), transcatheter aortic valve replacement (TAVR) is currently the treatment of choice in most patients with intermediate or high surgical risk [1,2] and is increasingly used in patients with lower surgical risk [3,4]. As a result, the numbers of TAVR procedures performed each year are steadily rising [5]. A concomitant increase in experience as well as technical advances helped to significantly reduce procedural complications within the last years. However, the incidence of conduction disturbances including bradycardia, left bundle branch block (LBBB), right bundle branch block (RBBB), and high grade atrio-ventricular block (HAVB) remains high. Permanent pacemaker implantation (PPI) following TAVR with self-expandable valves is frequent and occurs in 17–40% of patients [6,7,8]. In particular, patients treated with self-expandable valves and baseline conduction disturbances show a higher incidence of PPI after TAVR [7,9,10]. In addition, TAVR implantation depth is a critical procedural factor that determines the necessity of new pacemaker implantation [11].

Recently, a cusp-overlap view (COP) implantation technique has been proposed for the implantation of the self-expandable Core Valve Evolut valve in order to better visualize valve deployment to enhance implantation depth compared to the classical three-cusp view (TCV) [12,13]. Using the COP, the left ventricular outflow tract is elongated, so that the real implantation depth is visualized during valve deployment allowing for higher implantation. Additionally, COP accentuates the right-non commissure in the center of the fluoroscopic view which further allows for more precise valve implantation [13]. Two recent studies indicated lower pacemaker rates with comparable procedural and short-term risks after TAVR using the COP [11,14]. Nevertheless, superiority of the COP over the TCV remains uncertain and data addressing this topic are scarce. This study aimed to systematically analyze procedural and in-hospital outcomes after TAVR with self-expandable CoreValve Evolut prostheses using the COP compared to the TCV in a high-volume center.

## 2. Materials and Methods

### 2.1. Study Population

All consecutive patients undergoing transfemoral TAVR with a self-expandable CoreValve Evolut valve between April 2019 and October 2021 at the Munich University Hospital (Munich, Germany) were included in this analysis. Patients with prior permanent pacemaker or prior surgical or transcatheter aortic valve replacement were excluded. Considering previous reports suggesting an improvement of implantation depth with the modification of implant projection, COP was used in all 61 consecutive patients for the implantation of self-expandable Core Valve Evolut valves from December 2020 to October 2021 [12,15]. COP patients were compared to 61 consecutive patients receiving CoreValve Evolut prostheses from April 2019 to November 2020 using the traditional three-cusp view (TCV).

Prior to TAVR, a multidisciplinary heart team consensus by interventional cardiologists and cardiac surgeons was obligatory to evaluate the best treatment option in each individual patient. Patient data were collected and stored in a database according to the local requirements for quality control. Ethical approval was obtained from the institutional ethics board (EVERY-Valve-Registry, ethical code number 19-840; Date: 20 December 2019).

### 2.2. Echocardiography

Transthoracic echocardiographic images were obtained using the Philips EPIQ CVx prior to the TAVR procedure in accordance with current European and American guidelines [16,17]. Echocardiography was performed by experienced physicians and images and measurements were reviewed by an independent cardiologist of our center. The severity of AS was assessed using the continuity equation method. Before discharge, valve function including the presence of paravalvular leaks was evaluated as suggested by the recently published Valve Academic Research Consortium 3-criteria Guidelines (VARC-3) [18].

### 2.3. TAVR Procedure

**Figure 1:**
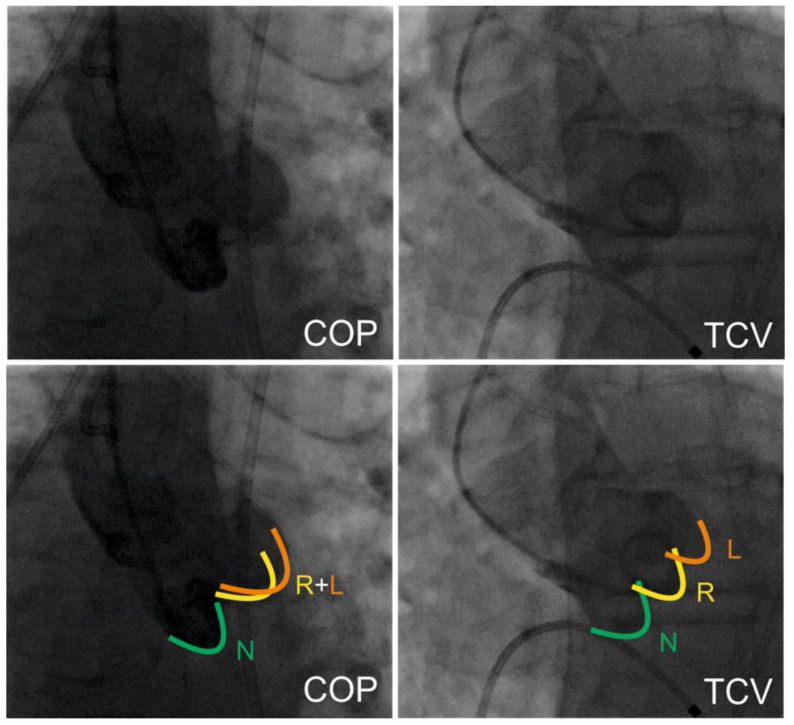
Implantation technique. This figure demonstrates the two different implantation techniques analyzed in this trial. COP (on the **left** side) with overlap of the right- and left coronary cusp and TCV (on the **right** side) with all three cusps visualized in line.

All procedures were performed under conscious sedation in combination with local anesthesia. Transfemoral access was used in all patients. Preprocedural anticoagulation was achieved with unfractionated heparin (50–70 IU/kg body weight). The decision to perform pre- and/or post-dilation was left to the operators’ discretion. The Cusp-Overlap view (COP) is defined as fluoroscopic overlap of the right (R, yellow) and left coronary cusp (L, orange) with an optimal visibility of the non-coronary cusp (N, green). In contrast, the right, left, and non-coronary cusps are visible in the classical Three-Cusp view. An overview on both fluoroscopic projections is given in Figure 1. For access-site hemostasis, suture-based and/or plug-based closure devices were used.

### 2.4. Trial Endpoints

The primary endpoint of this trial was defined as technical success according to the new 2021 VARC-3 criteria [18] and included absence of procedural mortality, device access and retrieval complications, false valve positioning, cardiac structural complications, and the need for multiple valve prostheses in one procedure.

Among secondary endpoints we defined cardiac structural complications including injury requiring surgery, pericardial effusion requiring intervention, coronary obstruction requiring intervention, in-hospital death, conversion to open surgery, and procedural stroke. Further secondary endpoints were the need for multiple valve prostheses in one procedure, paravalvular regurgitation >1+, conduction disturbances, permanent pacemaker implantation, bleeding, or vascular complications. Indications for PPI were high grade AV-block, progressive AV-block with additional new left bundle branch block and symptomatic persistent bradycardia according to current guideline recommendations [19]. The indications for PPI did not change throughout the study period.

### 2.5. Statistical Analysis

For descriptive statistics, continuous data are presented as means with standard deviation (SD) or medians with interquartile ranges [IQR], respectively. Categorical data are presented as proportions. Normality of data distribution was assessed graphically and using the Shapiro–Wilk test. Comparisons between groups were performed using the Chi-squared-test for categorical variables, Student’s *t*-test, or Mann–Whitney-U test for unpaired continuous variables and Wilcoxon rank sum test for paired variables according to data distribution. Logistic regression models with odds ratios and 95% confidence intervals (CI) were used for in-hospital outcome analysis.

A *p*-value of <0.05 was defined as statistically significant. The statistical software used for data analysis and visualization was R studio version 1.4.1717 (The R Foundation for Statistical Computing, Vienna, Austria).

## 3. Results

### 3.1. Baseline Characteristics

**Figure 2:**
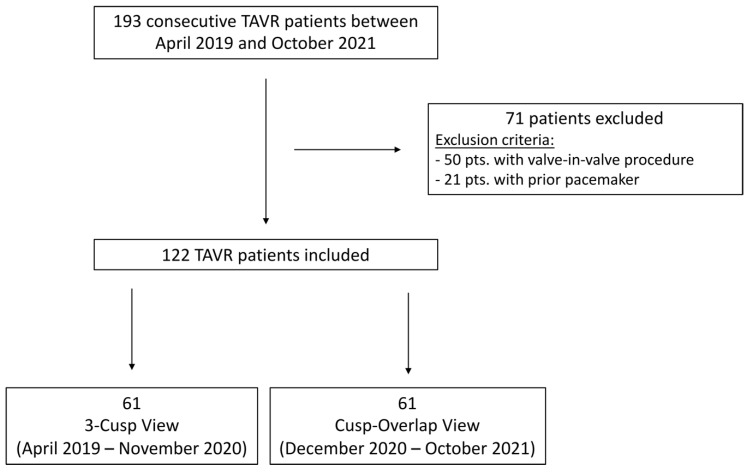
Flow chart study cohort. TAVR: transcatheter aortic valve replacement.

Out of the 193 consecutive patients who underwent TAVR with the CoreValve Evolut R prosthesis at our center between April 2019 and October 2021, a total of 122 patients with a median age of 83 [78, 87] years were included in this analysis. Seventy-one patients were excluded from the analysis, 50 patients with prior surgical bioprosthetic AVRs or prior TAVR and 21 patients with prior permanent pacemaker (Figure 2). Patients were considered to have elevated perioperative mortality by the local interdisciplinary heart team (median Society of Thoracic Surgeons (STS) score 3.3 [IQR: 2.2, 4.6]). All patients had severe symptomatic aortic stenosis and 86.0% reported advanced heart failure symptoms (NYHA functional class ≥III), (Table 1).

Out of 122 enrolled patients, TAVR prostheses were implanted using the traditional TCV in 61 patients, while in 61 patients the TAVR prostheses were implanted using the COP, respectively (Figure 2).

Overall baseline characteristics were comparable between groups. In particular mean pressure gradient and aortic valve area were comparable between TCV and COP patients (dpmean: 47.2 ± 17.4 mmHg vs. 41.7 ± 15.9 mmHg, *p* = 0.09; AVA: 0.64 ± 0.22 cm^2^ vs. 0.67 ± 0.19 cm^2^, *p* = 0.45). TCV patients had lower median LVEF compared to COP patients (58.0 [IQR: 52.0, 60.0%] vs. 60.0 [IQR: 56.5, 60.0%], *p* = 0.014, Table 1). Baseline electrocardiographic characteristics including prior RBBB (TCV: 6.7% vs. COP: 18.0%, *p* = 0.11) and prior AV Block (TCV: 11.7% vs. 6.6%, *p* = 0.5) were comparable between groups (Table 2). Computed tomography showed comparable rates of annulus calcification (TCV: 89.1% vs. COP: 73.6%, *p* = 0.07) and LVOT calcification (TCV: 1.8% vs. COP: 11.3%, *p* = 0.11). In particular, overall mean calcification score did not differ between groups (2507 ± 1444 AU vs. 2905 ± 1551 AU, *p* = 0.17) before TAVR. Additionally, there was no difference between baseline pharmacologic therapy including betablockers, amiodarone, ivabradine, digitalis, and calcium antagonists (Verapamil Type) (Supplementary Table S1).

### 3.2. Procedural and In-Hospital Outcome

All patients were treated with a Medtronic CoreValve Evolut R prothesis (Dublin, Ireland). Device sizes did not differ between groups (Table 2). Postprocedural maximal pressure gradient was 14.8 ± 8.2 mmHg vs. 14.3 ± 6.3 mmHg at discharge for TCV and COP, respectively.

**Figure 3:**
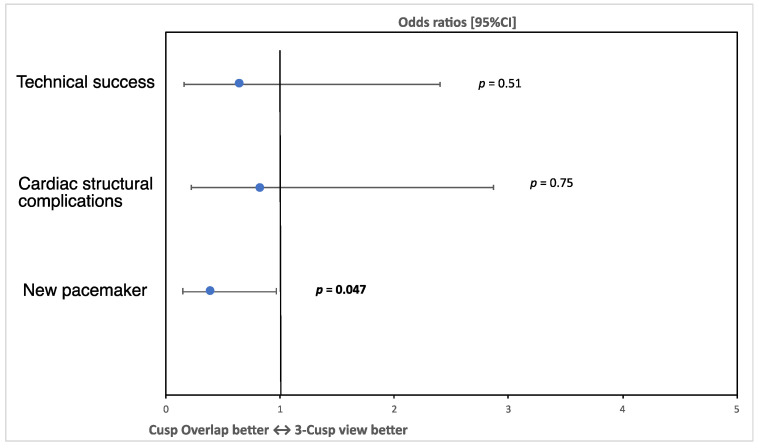
Primary and secondary endpoints: this figure displays the odds ratios and 95% confidence intervals for primary and secondary endpoints showing a significant difference regarding the risk for permanent pacemaker implantation.

There was no difference in the primary endpoint defined as technical success according to the VARC-3 definition [18] between TCV and COP patients (93.4% vs. 90.2% respectively, OR 0.65, 95% CI 0.16, 2.4, *p* = 0.51, Figure 3, Table 3). There was also no difference in cardiac structural complications (OR 0.82, 95% CI 0.22, 2,87, *p* = 0.75). However, there was a significantly lower risk for post-procedural permanent pacemaker implantation among COP compared to TCV patients (27.9% in the TCV group, 13.1% in the COP group, OR 0.39, 95% CI 0.15, 0.97, *p* = 0.047, Figure 3). The rate of postprocedural AR > 1+ did not differ significantly between both groups (TCV: 1.6% vs. COP: 4.9%, *p* = 0.61).

Bleeding and vascular complications were similar in both groups (bleeding: OR 0.47, 95% CI 0.096, 1.89, *p* = 0.31; vascular complications: OR 0.58, 95% CI 0.11, 2.47, *p* = 0.47). A detailed overview on procedural results according to the VARC-3 definition is shown in Table 3.

## 4. Discussion

This study demonstrates that the self-expandable CoreValve Evolut prosthesis can be implanted using the COP and the TCV with comparable efficacy and safety. However, valve deployment using the COP technique was associated with significantly lower risk of permanent pacemaker implantation after TAVR.

Despite the increase in experience and technology, the incidence of conduction disturbances requiring permanent pacemaker implantation (PPI) after TAVR with self-expandable valves remains high [6]. Unfortunately, modifiable procedural predictors for PPI after TAVR with self-expandable valves are rare [20]. A COP has been proposed for TAVR with self-expandable valves offering an improved visualization for a more precise valve deployment and recent studies indicated that this implantation technique is associated with a higher implantation depth [12,13].

In this context two recent studies analyzed the incidence of conductance disturbances in COP patients and reported lower rates of LBBB and PPI [11,21]. However, data on this topic are still limited while the clinical need to reduce the rate of PPI in these patients is high. This study analyzed a homogeneous cohort of consecutive TAVR patients treated with self-expandable valves in a high-volume center. We demonstrate that the application of the COP technique is safe and effective while the incidence of PPI could be reduced by over 50% from about 28% using the TCV to 13% using the COP. These results are in line with the current literature, where the rate of PPI was reduced to 11–13% using the COP [11,14]. In particular, in a recent propensity score-matched analysis, COP was associated with a higher implantation depth leading to a lower PPI-rate of 12% [14]. There was no difference in procedural complications such as significant aortic regurgitation or device dislocation. A major strength of this trial is the homogenous patient cohort with complete follow-up. It is important to mention that there were no procedural differences between both groups apart from the implantation technique. In particular, there were no differences in the rate of pre- and post-dilatation, the presence of LVOT-/leaflet-/annulus calcification or severity of aortic valve calcification measured by calcification score [22]. It is known that preprocedural conductance disturbances such as RBBB, LBBB or first-degree AV-Block are associated with augmented risk for PPI after TAVR with self-expandable valves [9,20]. These non-modifiable predictors were equally distributed between groups. Another strength of this study is the detailed analysis of these known risk factors for PPI after TAVR.

Some studies showed lower rates of new LBBB or RBBB using COP, which was not the case in our study. This may be caused by a rather small sample size. Nevertheless, COP patients showed a trend toward fewer permanent ECG changes after TAVR supporting the previously seen decrease in conduction disturbances.

Generally, the higher implantation depth that is desired when using the COP technique could possibly be associated with higher rates of device dislocation leading to further interventions due to significant aortic regurgitation. Pascual et al. reported that 3.5% of COP patients required a second valve implantation due to severe aortic valve regurgitation (AR) secondary to device dislocation [11]. In our study this rare event occurred in one patient in the COP group (1.6%) indicating a stable valve position using the COP. Despite these positive results, larger studies or meta-analyses with a longer follow-up are needed to confirm this important aspect to assure safety and efficacy of COP compared to TCV [13].

### Study Limitations

Several limitations have to be acknowledged and mostly derive from the retrospective nature of the study. Moreover, operators’ learning curve may have affected the PPI rate. However, enrollment period was short and treatment was performed by the same experienced interventionalists which should have reduced operators’ variability. Finally, our study was focused on one type of self-expandable valve and therefore, the results and conclusions should be interpreted in this context.

## 5. Conclusions

Implantation of the self-expandable CoreValve Evolut prosthesis using the COP might help to reduce the rate of pacemaker implantations following TAVR by enhancing the visualization of the left ventricular outflow tract during valve deployment. Further studies with a longer follow-up period are needed to clarify the role of this promising implantation technique. Especially as TAVR is increasingly used in younger patients with lower surgical risk, a reduction of PPI will be of particular importance in the future [3,4].

## Figures and Tables

**Table 1:** Baseline characteristics. This table demonstrates the baseline characteristics of the study cohort.

Baseline Characteristics				
	Overall	Three-Cusp View	Cusp-Overlap View	*p*-Value
*n*	122	61	61	
Age (years)	83.2 [79.8, 87.4]	83.3 [80.4, 87.9]	82.5 [79.0, 86.8]	0.15
Sex (female)	25 (20.5)	10 (16.4)	15 (24.6)	0.37
BMI	25.2 [22.8, 28.3]	25.0 [21.8, 27.4]	25.5 [23.3, 28.7]	0.11
STS Score	3.3 [2.2, 4.6]	3.1 [2.0, 4.9]	3.5 [2.6, 3.7]	0.94
NYHA functional class				0.92
NYHA II	14 (14.4)	7 (14.0)	7 (14.9)	
NYHA III	78 (80.4)	40 (80.0)	38 (80.9)	
NYHA IV	5 (5.2)	3 ( 6.0)	2 (4.3)	
AV dpmean (mmHg)	44.5 (16.8)	47.2 (17.4)	41.7 (15.9)	0.09
AV dpmax (mmHg)	69.6 (26.3)	74.1 (27.4)	65.0 (24.6)	0.07
AV opening area (cm^2^)	0.66 (0.21)	0.64 (0.22)	0.67 (0.19)	0.45
LVEF (%)	59.4 [55.0, 60.0]	58.0 [52.0, 60.0]	60.0 [56.5, 60.0]	0.01
TAPSE (mm)	22.18 (7.30)	22.37 (8.84)	21.98 (5.10)	0.80
Diabetes	20 (20.2)	10 (19.6)	10 (20.8)	>0.99
Coronary artery disease	50 (49.5)	22 (42.3)	28 (57.1)	0.20
Previous MI	5 (5.4)	1 (2.1)	4 (8.7)	0.35
Previous PCI	22 (22.0)	8 (15.7)	14 (28.6)	0.19
Previous CABG	8 (7.1)	4 (6.5)	4 (6.5)	>0.99
Atrial fibrillation	51 (44.7)	25 (41.7)	26 (48.1)	0.61
Prior cardiac surgery	10 (8.9)	5 (8.8)	5 (8.8)	0.73

Qualitative data are presented as *n* (%); Quantitative data are presented as median [IQR] or mean (SD); BMI, body mass index; STS, Society of Thoracic Surgeons; AV, aortic valve; dpmean, mean pressure gradient; dpmax, maximum pressure gradient; NYHA, New York Heart Association; LVEF, left ventricular ejection fraction; TAPSE, tricuspid annular plane systolic excursion; MI; myocardial infarction; PCI, percutaneous coronary intervention; CABG, coronary artery bypass graft.

**Table 2:** Device sizes as well as electrocardiographic and computertomographic characteristics of patients.

Characteristics Associated with PPI				
	Overall	Three-Cusp View	Cusp-Overlap View	*p*-Value
*n*	122	61	61	
Device size (mm)				0.40
23	13 (10.7)	8 (13.3)	5 (8.2)	
26	65 (53.7)	33 (55.0)	32 (52.5)	
29	41 (33.9)	19 (31.7)	22 (36.1)	
34	2 (1.7)	0 (0.0)	2 (3.3)	
Electrocardiography				
Prior LBBB	17 (14.0)	8 (13.3)	9 (14.8)	>0.99
Prior AV block	11 (9.1)	7 (11.7)	4 (6.6)	0.51
Prior RBBB	15 (12.4)	4 (6.7)	11 (18.0)	0.11
Prior Afib	51 (44.7)	25 (41.7)	26 (48.1)	0.61
Prior bradycardia	15 (12.3)	7 (11.5)	8 (13.1)	1.00
Computertomography				
Leaflet calcification	103 (95.4)	50 (90.9)	53 (100.0)	0.07
Annulus calcification	88 (81.5)	49 (89.1)	39 (73.6)	0.07
LVOT calcification	7 (6.5)	1 (1.8)	6 (11.3)	0.11
Calcification Score (AU)	2704.1 (±1504.1)	2507.2 (±1444.3)	2904.7 (±1550.7)	0.17

Qualitative data are presented as *n* (%); quantitative data are presented as mean (SD). LBBB: left bundle branch block; AV: atrio-ventricular; RBBB: right bundle branch block; Afib: atrial fibrillation; LVOT: left ventricular outflow tract, PPI: permanent pacemaker implantation.

**Table 3:** In-hospital VARC-3 endpoints. This shows procedural endpoints in COP and TCV patients.

In-Hospital VARC-3 Endpoints				
	All	Three-Cusp View	Cusp-Overlap View	*p*-Value
*n*	122	61	61	
Technical success	112 (91.8)	55 (90.2)	57 (93.4)	0.52
Technical failure	10 (8.2)	6 (9.8)	4 (6.6)	0.74
Procedural mortality	1 (0.8)	1 (1.6)	0 (0.0)	>0.99
Device access and retrival complications	5 (4.1)	3 (4.9)	2 (3.3)	>0.99
False valve positioning	1 (0.8)	0 (0.0)	1 (1.6)	>0.99
Surgery or cardiac structural complications	11 (9.0)	6 (9.8)	5 (8.2)	>0.99
Multiple devices	1 (0.8)	0 (0.0)	1 (1.6)	>0.99
Cardiac structural complications	11 (9.0)	6 (9.8)	5 (8.2)	>0.99
Injury requiring surgery	2 (1.6)	1 (1.6)	1 (1.6)	1.00
PE requiring intervention	2 (1.6)	1 (1.6)	1 (1.6)	1.00
Coronary obstruction requiring intervention	1 (0.8)	1 (1.6)	0 (0.0)	>0.99
Death in hospital	1 (0.8)	1 (1.6)	0 (0.0)	>0.99
Open Surgery	1 (0.8)	0 (0.0)	1 (1.6)	>0.99
Procedural Stroke	5 (4.1)	3 (4.9)	2 (3.3)	>0.99
Echocardiography				
AR > I°	4 (3.3)	1 (1.6)	3 (4.9)	0.61
Postprocedural AV dpmax (mmHg)	14.5 (7.3)	14.8 (8.2)	14.3 (6.3)	0.73
Electrocardiography				
New LBBB	56 (45.9)	27 (44.3)	29 (47.5)	0.86
New AV Block any Degree	38 (31.1)	20 (32.8)	18 (29.5)	0.85
new IVCD > 120 ms	2 (1.6)	1 (1.6)	1 (1.6)	1.00
Temp. Pacer	36 (29.8)	20 (32.8)	16 (26.2)	0.55
Regredient ECG changes	36 (42.9)	16 (39.0)	22 (51.2)	0.18
Other				
Bleeding	9 (7.4)	6 (9.8)	3 (4.9)	0.49
Vascular complications	8 (6.6)	5 (8.2)	3 (4.9)	0.72
Resuscitation	4 (3.3)	4 (6.6)	0 (0.0)	0.13

Qualitative data are presented as *n* (%); VARC-3, Valve Academic Research Consortium 3; AR, aortic regurgitation; LBBB, left bundle branch block; AV, aortic valve; IVCD, intraventricular conductance disturbance; ECG, electrocardiography; dpmax, maximum pressure gradient.

## Data Availability

Data available on request due to restrictions e.g., privacy or ethical. The data presented in this study are available on request from the corresponding author.

## Supplementary Materials

The following are available online at https://www.mdpi.com/article/10.3390/jcm11061561/s1, Table S1: Baseline pharmacologic therapy.
