# Supplementary material for: Transcatheter Aortic Valve Replacement with the Self-Expandable Core Valve Evolut Prosthesis Using the Cusp-Overlap vs. Tricusp-View
Source: J Clin Med. 2022 Mar 12;11(6):1561. doi: 10.3390/jcm11061561 (PMC8953752; doi:10.3390/jcm11061561)
Supplement: Supplementary file 1 [file jcm-11-01561-s001.zip › jcm-1541536-supplementary.pdf]

Supplementary Materials

Table S1. Baseline pharmacologic therapy.

	**All**	**Three-Cusp View**	**Cusp-Overlap View**	***p*-Value**
**N**	**122**	**61**	**61**	
Betablockers	78 (63.9)	38 (62.3)	40 (65.6)	0.85
Amiodarone	2 (1.6)	1 (1.6)	1 (1.6)	1.00
Digitalis	1 (0.8)	1 (1.6)	0 (0.0)	1.00
Ivabradine	1 (0.8)	1 (1.6)	0 (0.0)	1.00
Calcium Antagonists (Verapamil-Type)	1 (0.8)	1 (1.6)	0 (0.0)	1.00

Qualitative data are presented as *n* (%).
